# Subtly altered topological asymmetry of brain structural covariance networks in autism spectrum disorder across 43 datasets from the ENIGMA consortium

**DOI:** 10.1038/s41380-022-01452-7

**Published:** 2022-02-08

**Authors:** Zhiqiang Sha, Daan van Rooij, Evdokia Anagnostou, Celso Arango, Guillaume Auzias, Marlene Behrmann, Boris Bernhardt, Sven Bolte, Geraldo F. Busatto, Sara Calderoni, Rosa Calvo, Eileen Daly, Christine Deruelle, Meiyu Duan, Fabio Luis Souza Duran, Sarah Durston, Christine Ecker, Stefan Ehrlich, Damien Fair, Jennifer Fedor, Jacqueline Fitzgerald, Dorothea L. Floris, Barbara Franke, Christine M. Freitag, Louise Gallagher, David C. Glahn, Shlomi Haar, Liesbeth Hoekstra, Neda Jahanshad, Maria Jalbrzikowski, Joost Janssen, Joseph A. King, Luisa Lazaro, Beatriz Luna, Jane McGrath, Sarah E. Medland, Filippo Muratori, Declan G. M. Murphy, Janina Neufeld, Kirsten O’Hearn, Bob Oranje, Mara Parellada, Jose C. Pariente, Merel C. Postema, Karl Lundin Remnelius, Alessandra Retico, Pedro Gomes Penteado Rosa, Katya Rubia, Devon Shook, Kristiina Tammimies, Margot J. Taylor, Michela Tosetti, Gregory L. Wallace, Fengfeng Zhou, Paul M. Thompson, Simon E. Fisher, Jan K. Buitelaar, Clyde Francks

**Affiliations:** 1 Department of Language & Genetics, Max Planck Institute for Psycholinguistics, Nijmegen, The Netherlands; 2 Department of Cognitive Neuroscience, Donders Institute for Brain, Cognition and Behaviour, Donders Centre for Cognitive Neuroimaging, Radboud University Medical Centre, Nijmegen, The Netherlands; 3 Bloorview Research Institute, Holland Bloorview Kids Rehabilitation Hospital and Department of Pediatrics, University of Toronto, Toronto, ON Canada; 4 Child and Adolescent Psychiatry Department, Institute of Psychiatry and Mental Health, Gregorio Maran General University Hospital, School of Medicine, Universidad Complutense, IiSGM, CIBERSAM, Madrid, Spain; 5 Institut de Neurosciences de la Timone, UMR 7289, Aix Marseille Universit, CNRS, Marseille, France; 6 Department of Psychology and Neuroscience Institute, Carnegie Mellon University, Pittsburgh, PA USA; 7 McConnell Brain Imaging Centre, Montreal Neurological Institute and Hospital, McGill University, Montreal, QC Canada; 8 Center of Neurodevelopmental Disorders (KIND), Centre for Psychiatry Research; Department of Women’s and Children’s Health, Karolinska Institutet & Stockholm Health Care Services, Region Stockholm, Stockholm, Sweden; 9 Child and Adolescent Psychiatry, Stockholm Health Care Services, Region Stockholm, Stockholm, Sweden; 10 Curtin Autism Research Group, Curtin School of Allied Health, Curtin University, Perth, WA Australia; 11 Laboratory of Psychiatric Neuroimaging (LIM-21), Departamento e Instituto de Psiquiatria, Hospital das Clinicas HCFMUSP, Faculdade de Medicina, Universidade de Sao Paulo, Sao Paulo, SP Brazil; 12 IRCCS Stella Maris Foundation, Pisa, Italy; 13 Department of Clinical and Experimental Medicine, University of Pisa, Pisa, Italy; 14 Department of Child and Adolescent Psychiatry and Psychology Hospital Clinic, Psychiatry Unit, Department of Medicine, 2017SGR881, University of Barcelona, IDIBAPS, CIBERSAM, Barcelona, Spain; 15 Department of Forensic and Neurodevelopmental Sciences, Institute of Psychiatry, Psychology & Neuroscience King’s College London, London, UK; 16 BioKnow Health Informatics Lab, College of Computer Science and Technology, and Key Laboratory of Symbolic Computation and Knowledge Engineering of Ministry of Education, Jilin University, Changchun, Jilin, 130012 China; 17 Brain Center Rudolf Magnus, Department of Psychiatry, University Medical Center Utrecht, Utrecht, The Netherlands; 18 Department of Child and Adolescent Psychiatry, Psychosomatics and Psychotherapy, University Hospital, Goethe University Frankfurt am Main, Frankfurt, Germany; 19 The Sackler Institute for Translational Neurodevelopment, Institute of Psychiatry, Psychology & Neuroscience, King’s College London, London, UK; 20 Department of Child and Adolescent Psychiatry & Division of Psychological and Social Medicine and Developmental Neurosciences, Faculty of Medicine, TU Dresden, Dresden, Germany; 21 Institute of Child Development, Department of Pediatrics, Masonic Institute of the Developing Brain, University of Minnesota, Minneapolis, MN USA; 22 Department of Psychiatry, University of Pittsburgh School of Medicine, Pittsburgh, PA USA; 23 Department of Psychiatry, School of Medicine, Trinity College, Dublin, Ireland; 24 The Trinity College Institute of Neuroscience, Trinity College, Dublin, Ireland; 25 Department of Human Genetics, Donders Institute for Brain, Cognition and Behaviour, Radboud University Medical Center, Nijmegen, The Netherlands; 26 Department of Psychiatry, Donders Institute for Brain, Cognition and Behaviour, Radboud University Medical Center, Nijmegen, The Netherlands; 27 Department of Psychiatry, Boston Children’s Hospital and Harvard Medical School, Boston, MA 02115-5724 USA; 28 Olin Neuropsychiatric Research Center, Hartford, CT USA; 29 Department of Brain Sciences, Imperial College London, London, UK; 30 Karakter Child and Adolescent Psychiatry University Centre, Nijmegen, The Netherlands; 31 Imaging Genetics Center, Mark and Mary Stevens Neuroimaging and Informatics Institute, Keck School of Medicine of USC, University of Southern California, Marina del Rey, CA USA; 32 Psychiatric Genetics, QIMR Berghofer Medical Research Institute, Brisbane, QLD Australia; 33 Behavioural Genetics Clinic, Adult Autism Service, Behavioural and Developmental Psychiatry Clinical Academic Group, South London and Maudsley Foundation NHS Trust, London, UK; 34 Department of Physiology and Pharmacology, Wake Forest Baptist Medical Center, Winston-Salem, NC USA; 35 Magnetic Resonance Image Core Facility, IDIBAPS (Institut d’Investigacions Biomdiques August Pi i Sunyer), Barcelona, Spain; 36 National Institute for Nuclear Physics, Pisa Division, Largo B. Pontecorvo 3, Pisa, Italy; 37 Institute of Psychiatry, King’s College London, London, UK; 38 Astrid Lindgren Children’s Hospital, Karolinska University Hospital, Region, Stockholm, Sweden; 39 Center of Neurodevelopmental Disorders (KIND), Centre for Psychiatry Research; Department of Womens and Childrens Health, Karolinska Institutet and Child and Adolescent Psychiatry, Stockholm Health Care Services, Stockholm County Council, Stockholm, Sweden; 40 Diagnostic Imaging, The Hospital for Sick Children, and Department of Medical Imaging, University of Toronto, Toronto, ON Canada; 41 Department of Speech, Language, and Hearing Sciences, The George Washington University, Washington, DC USA; 42 Donders Institute for Brain, Cognition and Behaviour, Radboud University, Nijmegen, The Netherlands

**Keywords:** Autism spectrum disorders, Psychiatric disorders

## Abstract

Small average differences in the left-right asymmetry of cerebral cortical thickness have been reported in individuals with autism spectrum disorder (ASD) compared to typically developing controls, affecting widespread cortical regions. The possible impacts of these regional alterations in terms of structural network effects have not previously been characterized. Inter-regional morphological covariance analysis can capture network connectivity between different cortical areas at the macroscale level. Here, we used cortical thickness data from 1455 individuals with ASD and 1560 controls, across 43 independent datasets of the ENIGMA consortium’s ASD Working Group, to assess hemispheric asymmetries of intra-individual structural covariance networks, using graph theory-based topological metrics. Compared with typical features of small-world architecture in controls, the ASD sample showed significantly altered average asymmetry of networks involving the fusiform, rostral middle frontal, and medial orbitofrontal cortex, involving higher randomization of the corresponding right-hemispheric networks in ASD. A network involving the superior frontal cortex showed decreased right-hemisphere randomization. Based on comparisons with meta-analyzed functional neuroimaging data, the altered connectivity asymmetry particularly affected networks that subserve executive functions, language-related and sensorimotor processes. These findings provide a network-level characterization of altered left-right brain asymmetry in ASD, based on a large combined sample. Altered asymmetrical brain development in ASD may be partly propagated among spatially distant regions through structural connectivity.

## Introduction

Autism spectrum disorder (ASD) is a childhood-onset condition of neurodevelopmental origin with a prevalence of roughly 1% [1–4]. Individuals with ASD are characterized by social communication and interaction challenges alongside restricted and/or repetitive behaviors causing functional impairment in major areas of life [3]. Language delay is also a common feature of the disorder [5, 6].

Brain regions important for social cognition and language show lateralized activation in functional neuroimaging studies, in the majority of people [7]. For example, roughly 90% of the adult population has left-hemispheric dominance for word generation tasks, which particularly elicit activation of inferior frontal and temporal cortex [8, 9], while theory-of-mind tasks typically elicit rightward asymmetrical activation around the temporo-parietal junction [10]. Various studies have indicated that these functional asymmetries can be altered in ASD [11]. Using functional magnetic resonance imaging (fMRI), increased autism symptom severity and ASD case-control status have been associated with reduced laterality of activation or inter-regional connectivity during language and social cognition tasks [12–14]. Positron emission tomography has also identified reduced frontal activation of the left hemisphere during sentence processing in adults with ASD [15]. Magnetoencephalography has revealed altered maturational changes of the laterality of cortical electrophysiology in children with ASD, in response to passive auditory presentation of vowel stimuli [16]. In terms of brain structure, altered asymmetries of regions of the cortex important for language and/or social cognition, including lateral temporal regions and the fusiform gyrus [17–19], have been reported in individuals with ASD. In addition, an increased rate of non-right-handedness–a behavioral trait linked to brain asymmetry [20]–has been found in individuals with ASD, including by meta-analysis across studies [21, 22]. These findings suggest that altered asymmetrical neurodevelopment is linked etiologically to ASD behavioral characteristics.

We recently performed the largest-to-date study of brain structural asymmetry in ASD [17], analyzing a total of 1774 affected individuals and 1809 controls from multiple datasets made available by the ASD working group of the international ENIGMA (Enhancing Neuro-Imaging Genetics through Meta-Analysis) consortium [23, 24]. ASD was most notably associated with widespread alterations of cortical thickness asymmetry, involving the medial frontal, posterior cingulate and inferior temporal cortex. These regions overlapped with those showing altered functional lateralization for language and social cognitive tasks in ASD [12, 13].

The widespread nature of altered cortical thickness asymmetries in ASD, over multiple non-contiguous regions, raises new questions: is there altered asymmetry of topological network organization in ASD, and if so, which specific cortical regions are involved in the affected structural networks? Network organization can be investigated using cortical thickness data from in vivo, non-invasive structural MRI, by studying the inter-regional covariance of thickness measures, as has been performed previously in data from people with other disorders, or else unaffected people [25–28]. Cortical thickness is a widely-used morphological measure to estimate structural networks of this type [29], as it relates to underlying features such as the sizes and densities of neurons [30, 31], as well as functional and white matter connectivity [29, 32]. While it is not fully understood how inter-regional covariation of cortical thickness arises, one prevailing hypothesis is that synapses can have mutually trophic and protective effects on the pre- and post-synaptic neurons involved, such that increased inter-regional connectivity can lead to co-variance at the macro-anatomical level [26]. In addition, synchronous firing between neurons could trigger coordinated synaptogenesis and growth of more highly connected regions [33, 34].

Neural connections may also propagate pathological processes between spatially distant regions [35], which has led to a notion of brain disorders as being partly “disconnection syndromes” [36–38]. For example, lower structural covariance based on regional thickness measures from the fronto-temporal cortex has been observed in individuals with ASD relative to typically developing controls, an association which may also be modulated by language and social cognitive abilities [39–42]. However, the regions in these studies were defined by *prior* knowledge of language, whereas alterations of cortical thickness in ASD are more widespread than this [23]. Transcriptome analyzes based on postmortem cortical tissue have implicated disrupted biological pathways affecting cell number, cortical patterning and differentiation, axon guidance, synaptic activity and plasticity-related processes in ASD [43, 44]. This also suggests a broader impact on cortical structure beyond core language regions.

The structural connectivity between two cortical regions can be derived from Pearson correlation between their thickness measures, as calculated across all individuals within a given group (such as cases or controls) [25, 26, 45]. However, this cross-subject approach only produces group-level connectivity measures. An alternative approach, which we adopted here, is to measure intra-individual structural covariance, i.e. the structural covariance between different brain regions within each individual. This approach captures global and regional network characteristics at an individual level, and has been applied previously to various psychiatric and neurological disorders [27, 28, 46] (see below for details of the method). Importantly, as the intra-individual approach permits the derivation of individualized topological measures, it can be used to examine associations with clinical variables in individuals with ASD, as well as examining case-control, group average effects.

Thus far, investigations of altered topological network connectivity in ASD have been impeded by limited sample sizes in relation to subtle effects, and the likely neurobiological heterogeneity of ASD. In addition, no previous studies of structural covariance network connectivity in ASD have addressed the possibility of altered network left-right asymmetry at the whole-hemisphere level. Here, we hypothesized that ASD is associated with subtle average reorganization of hemispheric cortical thickness covariance network architecture, such that altered inter-regional connectivity asymmetry could link some of the disparate regions that have previously shown altered asymmetry in separate region-by-region testing [17]. We used structural MRI data from 43 datasets (1455 ASD patients and 1560 unaffected controls), collected by members of the ENIGMA consortium’s ASD Working Group, to perform the first graph-based, cortex-wide analysis of structural covariance network asymmetry in ASD. This was followed by functional annotation of affected networks through the use of meta-analyzed functional neuroimaging data, as well as tests relating altered structural network covariance asymmetry within ASD individuals to symptom severity, psychiatric medication use, IQ, age, sex and handedness.

## Material and Methods

### Datasets and participants

Structural T1-weighted brain MRI-derived data were available via the ENIGMA-ASD Working Group [23]. After data quality control (see below), there were 1455 individuals with ASD (mean age: 15.65 years, range 2–64 years, 1213 males) and 1560 healthy controls (mean age: 16.09 years, range 2–64 years, 1179 males) across 43 separate datasets (Table 1). Clinical diagnosis of ASD was made according to DSM-IV criteria. Binary categorical data on handedness were available for 599 ASD individuals (551 right-handed, 48 left-handed). See Supplementary Methods for further information on participants and assessments. For each of the data sets, all relevant ethical regulations were complied with, and appropriate informed consent was obtained for all individuals.

**Table 1:** Characteristics of the 43 datasets of the ENIGMA Autism Spectrum Disorder working group that were used in this study.

Dataset no.	Dataset name	*N* total	*N* cases (M/F)	*N* controls (M/F)	Mean age (min, max)	Scanner type	Field strength
1	ABIDE_CALTECH	31	13/1	13/4	29.1 (17.5, 56.2)	Siemens Trio	3 T
2	ABIDE_LEUVEN_2	35	12/3	15/5	14.2 (12.1, 16.9)	Philips Interna	3 T
3	ABIDE_MAX_MUN	51	21/3	24/3	26.5 (7, 58)	Siemens Verio	3 T
4	ABIDE_NYU	184	68/10	81/25	15.3 (6.5, 39.1)	Siemens Allegra	3 T
5	ABIDE_OLIN	36	17/3	14/2	16.8 (10, 24)	Siemens Allegra	3 T
6	ABIDE_PITT	58	26/5	23/4	19.2 (9.3, 35.2)	Siemens Allegra	3 T
7	ABIDE_SBL	30	15/0	15/0	34.4 (20, 64)	Philips Interna	3 T
8	ABIDE_SDSU	37	14/1	16/6	15.0 (8.7, 37.7)	GE MR750	3 T
9	ABIDE_STANFORD	40	16/4	16/4	10.0 (7.5, 12.9)	GR Signa	3 T
10	ABIDE_TCD	55	24/1	30/0	16.7 (9.3, 25.9)	Philips Achieva	3 T
11	ABIDE_UM_1	126	48/14	41/23	12.8 (8.1, 20.9)	GE Signa	3 T
12	ABIDE_UM_2	31	14/1	15/1	15.3 (11.1, 26.8)	GE Signa	3 T
13	ABIDE_USM	100	59/0	41/0	21.3 (8.2, 50.2)	Siemens Trio	3 T
14	ABIDE_YALE	55	20/8	19/8	12.7 (7, 17.8)	Siemens Magnetom	3 T
15	ABIDEII-BNI	57	28/0	29/0	38.1 (18, 6)	Philips Ingenia	3 T
16	ABIDEII-EMC	41	18/2	19/2	8.3 (6.4, 10.7)	GE MR750	3 T
17	ABIDEII-ETH	31	11/0	20/0	22.9 (13.8, 30.7)	Philips Achieva	3 T
18	ABIDEII-GU	98	39/8	26/25	10.7 (8.1, 13.9)	Siemens TriTim	3 T
19	ABIDEII-IP	52	14/7	9/22	20.7 (6.1, 46.6)	Siemens TriTim	1.5 T
20	ABIDEII-IU	39	15/4	15/5	24.4 (17, 54)	Philips Achieva	3 T
21	ABIDEII-KKI	199	35/15	94/55	10.3 (8.0, 13.0)	Philips Achieva	3 T
22	ABIDEII-NYU_1	72	38/4	28/2	10.1 (5.2, 34.8)	Siemens Allegra	3 T
23	ABIDEII-OHSU	92	29/7	27/29	11.0 (7, 15)	Siemens Skyra	3 T
24	ABIDEII-OILH	39	12/1	17/9	23.5 (18, 31)	Siemens TriTim	3 T
25	ABIDEII-SDSU	57	26/7	22/2	13.0 (7.4, 18)	GE MR750	3 T
26	ABIDEII-TCD	41	19/0	22/0	15.4 (10, 20)	Philips Achieva	3 T
27	ABIDEII-USM	32	15/2	12/3	21.4 (9.1, 38.9)	Siemens TriTim	3 T
28	BRC	44	17/0	27/0	14.8 (10, 18)	GE Signa HDx	3 T
29	Barcelona	52	29/2	20/1	12.0 (7.3, 17.1)	Siemens Trio	3 T
30	Dresden	45	18/3	20/4	35.3 (21.1, 56.8)	Siemens Trio	3 T
31	FAIR	81	33/6	27/15	11.5 (7.8, 15.9)	Siemens Magnetom	3 T
32	FSM	80	20/20	20/20	4.1 (1.8, 6)	GE Signa	1.5 T
33	MRC	137	67/0	70/0	27.0 (18, 45)	GE Signa HDx	3 T
34	PITT_1	56	11/3	34/8	16.3 (8, 36)	Siemens Allegra	3 T
35	PITT_2	89	38/6	39/6	17.0 (8, 36)	Siemens Allegra	3 T
36	ParelladaHGGM	66	33/2	30/1	12.5 (7, 18)	Philips Intera	1.5 T
37	TCD_2	27	10/0	17/0	16.9 (12.7, 24.8)	Philips Achieva	3 T
38	TORONTO_1	177	70/20	45/42	11.8 (3.3, 20.8)	Siemens Trio	3 T
39	TORONTO_2	192	99/41	28/24	11.0 (2.5, 21.7)	Siemens Trio	3 T
40	UMCU_1	57	25/3	27/2	14.3 (7.1, 24.7)	Philips	1.5 T
41	NIJMEGEN2	68	27/18	15/8	26.3 (18, 40)	Siemens Avanto	1.5 T
42	NIJMEGEN3	92	36/4	43/9	9.5 (6.1, 12.3)	Siemens Avanto	1.5 T
43	NIJMEGEN1	33	14/3	14/2	15.0 (12.3, 18.0)	Siemens Trio	3 T

### Image acquisition and processing

Structural T1-weighted brain MRI scans were collected at each separate study site, using a variety of different scanners and protocols at field strengths of either 1.5 or 3 T (Table 1). Following this heterogeneous image acquisition, all sites applied the same harmonized protocol from the ENIGMA consortium for data processing and quality control [23, 24]. FreeSurfer [47] (version 5.3) was used to derive mean cortical thickness measures for each of 68 cortical regions (34 per hemisphere) defined by the Desikan–Killiany atlas [48]. See Supplementary Methods for more information on MRI processing and quality control. Freesurfer segmentation has been validated in data from age ranges as young as preschoolers [49], where it was found to be of generally good quality even before visual quality control of the type applied here.

### Construction of intra-individual hemispheric structural covariance networks

**Fig. 1:**
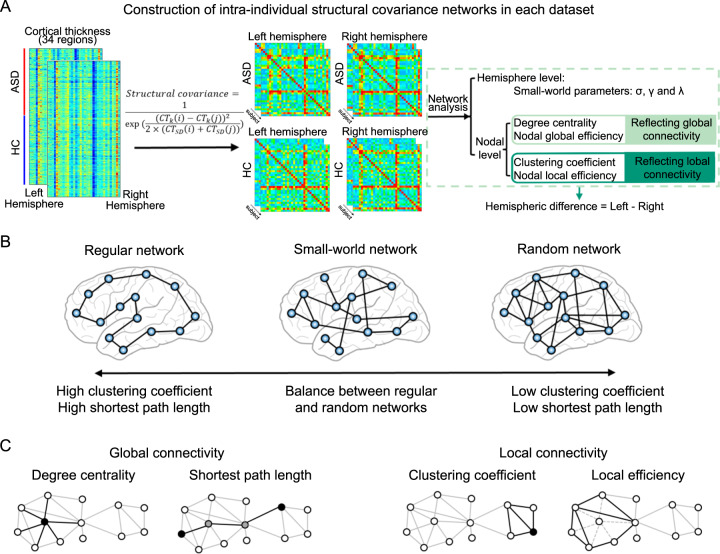
Schematic workflow of this study. **A** Flowchart of the procedure used in the current study. We first constructed intra-individual, intra-hemispheric structural covariance networks in each dataset using regional cortical thickness data. Then, for each individual, we computed graph theory metrics at the global and nodal levels using the intra-hemispheric networks. Finally, we calculated individual-level hemispheric differences for each metric, to examine case-control differences of topological network asymmetry. **B** Small-world network model. At the whole-hemisphere level, we estimated network integration and segregation using small-world parameters. A regular network is characterized by a high clustering coefficient and long shortest path length, corresponding to high local specialization and low global integration. In contrast, a random network has a low clustering coefficient and short shortest path length, corresponding to low local specialization and greater global integration. A small-world model reflects a balance between the extremes of local specialization versus global integration. **C** At the nodal level, we examined four graph theory measures: degree centrality and nodal global efficiency both measure global connectivity from/to a given node, whereas the cluster coefficient and nodal local efficiency reflect local connectivity from/to that node. Abbreviations: ASD autism spectrum disorder; HC healthy control; SD standard deviation.

Within each dataset, regional cortical thickness values were used to separately construct left-hemispheric and right-hemispheric structural covariance networks for each individual (Fig. 1), following an approach introduced and/or applied elsewhere [27, 28]. Such networks are comprised of nodes and edges, in which each cortical region represents a node. The edge between each pair of regions in a given individual was calculated with respect to the standard deviations for those regional measures calculated from control individuals (see Supplementary Methods for the formula) [27, 28]. For each individual, this approach yielded two separate 34 × 34 matrices, one for the left hemisphere and one for the right hemisphere, each representing a network of intra-hemispheric structural connectivity with 561 edges. We then removed weak connectivity with a sparsity threshold of *S* = 0.4, separately for each individual and network. Specifically, only the top 40% of strongest edges (that is, 224 edges) within any hemispheric network of any individual were retained–these were given the value 1, and the remainder (the weaker 60% of edges) were given the value 0 [50]. This approach ensured that all hemispheric structural networks of all individuals had the same number of edges, to explore case-control differences with respect to network measures. The sparsity threshold was also varied in sensitivity analyzes, see further below.

### Hemisphere-level network properties

We used the Brain Connectivity Toolbox [51] and GRETNA [52] toolbox to calculate topological network indices (Fig. 1). At the whole-hemisphere level, we calculated small-world parameters to measure the balance between network integration and segregation, as previously described [25, 53–55]. These procedures resulted in three hemisphere-level connectivity metrics for each individual and hemispheric network: the normalized clustering coefficient γ, the normalized shortest path length λ, and the small-world index σ (see Supplementary Methods for formulas and further explanation).

### Node-level network properties

For each of the 34 nodes, separately per individual and hemisphere, we calculated four measures which have been used in previous studies exploring brain topological network differences between individuals with ASD and controls (without investigating asymmetry) [56, 57]: the degree centrality and nodal global efficiency (both indicate connectivity globally from/to a given node) and the clustering coefficient and nodal local efficiency (both indicate local connectivity from/to a given node) [50, 58, 59]. Note that nodal global efficiency, i.e., the global efficiency of a given node, measures the connectivity linking that node to all other nodes (see Supplementary Methods for formulas and further explanation).

### Hemispheric asymmetry

To quantify the asymmetry of each separate network metric within each individual, we calculated the hemispheric difference (HD):

$${\it{HD}} = {\it{Left}} - {\it{Right}}$$

Therefore, a positive value of HD represents a leftward asymmetry for a given metric, while a negative HD represents a rightward asymmetry for that metric. (Note that the widely-used asymmetry index (Left-Right)/(Left+Right) would be less well suited to the present study, as Left and/or Right could sometimes take the value zero for the metrics defined above, in which case this index would take extreme or undefined values [60].)

### Statistical analysis

We used linear mixed effects random-intercept models (“fitlme” function in MATLAB version 2016a (The Mathworks Inc.)) to test for case-control differences, across all datasets simultaneously, but separately for each network metric HD. All models included the same fixed effects, i.e., diagnosis (case versus control status), age and sex, plus a random effect indicating which of the 43 datasets an individual was from, as shown in the following formula:

$${\it{HD}} = {\it{diagnosis}} + {\it{age}} + {\it{sex}} + {\it{random}}\,{{{{{{{\mathrm{(}}}}}}}}{\it{dataset}}{{{{{{{\mathrm{)}}}}}}}}$$

The random effect “dataset” adjusted for all variables that differed between datasets, including scanner type and field strength (Table 1). The *t* values derived from the “diagnosis” factor were used to compute Cohen’s *d* effect sizes for case-control difference effects [61]. Empirical *p* values were determined by 10,000 label swapping permutations (Supplementary Methods). For the 3 network-level metric HDs, significance was determined through the empirical *p* values for case-control effects with Bonferroni correction of 0.05/3. For the node-level network metric HDs, significance was determined for case-control effects using false discovery rate (FDR) correction for 34 nodes, with threshold pFDR < 0.05/4 (due to testing four node-level network measures).

### Directions of topological network asymmetry changes

For any HDs showing significant case-control differences in the main analysis above, we used linear mixed effects models to examine separately the corresponding left and right metrics to understand the unilateral effects. Models with the same fixed and random effects were used as above, again with 10,000 permutations. As this was a post hoc analysis to further describe any specific alterations of asymmetry in cases, we did not perform multiple testing correction for these analyses.

### Associations with ASD severity, medication, IQ, age, sex or handedness

For each topological network HD that showed significant associations with case-control status in the main analysis, we used separate linear mixed effects models to examine possible relationships between these HDs and ASD severity (total ADOS scores), current psychiatric medication use, IQ, age, sex or handedness within the ASD individuals only. These analyses would inform whether major aspects of case heterogeneity were related to the relevant topological network HDs. See Supplementary Methods for the models, sample sizes for each variable, and significance determination.

### Descriptive edge-level analysis

For each specific node that showed a significant case-control difference of degree centrality asymmetry in the main analysis, we extracted the intra-hemispheric structural connectivity values (i.e., one value for each edge) linking this “seed” node to all the 33 other nodes, separately from each hemispheric structural covariance network of each individual (this time without thresholding and binarization for sparsity, see above). For each matched pair of left and right edges, we then calculated the HD (again as Left-Right). The same linear mixed effects random-intercept model as the main analysis was used to examine each edge HD as the dependent measure across individuals, and 10,000 permutations were again used to assess the empirical two-tailed significance of the effect of diagnosis. Separately for each relevant node, the *p* value was FDR-corrected at 0.05 for multiple testing over the 33 edges connecting to that node.

### Cognitive functional annotation based on Neurosynth

To indicate the potential cognitive functions of regions that showed altered degree centrality asymmetry, we used the online platform Neurosynth [62] (https://neurosynth.org/) which includes meta-analytic brain maps based on input data from > 14,000 human functional neuroimaging studies. As of February 2021 there were 1307 maps in the database, representing different terms that capture diverse cognitive functions. Each map indicates a pattern of brain activation linked to a given term, through semantically-related words that occurred in the papers describing those studies. The large size of the database has been shown to compensate for any imperfect assignment of activations to particular cognitive domains or tasks [62]. This approach therefore provides a data-driven alternative to assigning brain regional functions by ad hoc, selective citations of limited numbers of papers from the literature.

Separately for each cortical region with significantly altered asymmetry of degree centrality in the node-level analysis, plus all regions linked to them by edges that showed significant alterations of asymmetry in ASD in the edge-level analysis (see above), we labeled these regions in both hemispheres to generate a bilateral mask. Then, we applied the “mri_surf2vol” function in FreeSurfer to project the surface-based brain mask into MNI152 standard volume space. The resultant volume-based binary masks were then used as input to identify region-associated cognitive terms through the Neurosynth “decoder” function. Finally, cognitive terms with correlations > 0.2 were visualized on a word-cloud plot, with sizes scaled according to their correlations with the corresponding meta-analytic maps generated by Neurosynth, while excluding anatomical terms, non-specific terms (e.g. ‘Tasks’), and one from each pair of virtually duplicated terms (such as “Words” and “Word”).

### Sensitivity analyses

To assess robustness with respect to the sparsity threshold 0.4 that was used in the main analysis, we repeated the analyses under varying sparsity thresholds ranging from 0.25 to 0.5 (with an interval of 0.01), and performed an area-under-the-curve analysis across this range (Supplementary Methods). Outside of the sparsity range 0.25–0.5 the defined networks are expected to lose connectedness and/or small-world organization [50].

To assess whether non-linear age could have an impact on case-control differences of network HDs, we repeated the main analysis including a non-linear age term (age-mean_age) [2] as a fixed effect (Supplementary Methods).

To assess whether results were affected by the global average cortical thickness of individuals, we reran the main analysis but included an extra fixed effect to represent average cortical thickness over all regions (Supplementary Methods).

## Results

### Hemisphere-level network asymmetries

None of the three hemisphere-level metric HDs (i.e., the normalized clustering coefficient γ, the small-world index σ, or the normalized shortest path length λ) showed significant differences between individuals with ASD and controls (all *p* > 0.05). A non-significant trend effect of diagnosis was observed for a leftward shift in λ asymmetry in ASD (Cohen’s *d* = 0.06, *p* = 0.10; Supplementary Table 1). Unilateral analysis of each hemisphere showed that ASD was nominally associated with reduced λ in the right hemisphere (Cohen’s *d* = −0.07, unadjusted *p* = 0.04), but not in the left hemisphere (Cohen’s *d* = 0.004, *p* = 0.92), which hints at a more efficient global information transmission and a shift towards randomization of right hemisphere networks in ASD (Supplementary Table 2).

### Node-level network measures

**Fig. 2:**
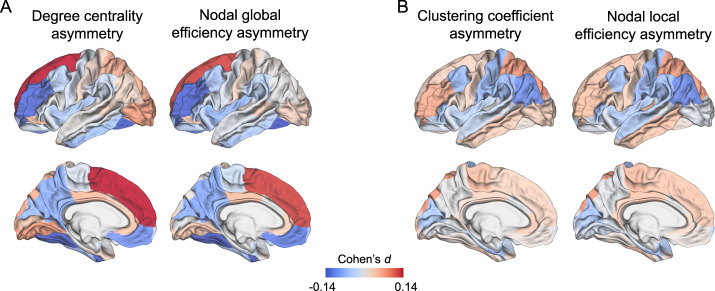
Cohen’s *d* effect sizes of ASD case-control associations for node-level topological asymmetries. **a** Effect sizes from ASD case-control analysis of node-level topological metric asymmetries that reflect global connectivity of each node, i.e., degree centrality and nodal global efficiency. **b** Effect sizes from ASD case-control analysis of nodal-level topological metric asymmetries that reflect local connectivity of each node, i.e., the clustering coefficient and nodal local efficiency. Positive effect sizes (pink-red) indicate shifts towards greater leftward or reduced rightward asymmetry in ASD compared to controls, and negative effect sizes (blue) represent shifts towards greater rightward asymmetry or reduced leftward asymmetry in ASD compared to controls.

We mapped the Cohen’s *d* effect sizes of associations between node-level network measure HDs and ASD over the whole cerebral cortex (Fig. 2). Effect sizes were low, ranging from −0.15 (nodal global efficiency HD of fusiform) to 0.14 (degree centrality HD of superior frontal cortex) (Supplementary Tables 3–6). Among node-level metric HDs, the degree centrality asymmetries of three regions, namely fusiform (Cohen’s *d* = −0.14, *p* < 0.0001), rostral middle frontal cortex (Cohen’s *d* = −0.13, *p* = 0.0007) and superior frontal cortex (Cohen’s *d* = 0.14, *p* = 0.0003), were significantly associated with ASD after FDR correction (Fig. 2 and Supplementary Table 3). In addition, nodal global efficiency HDs of four regions, namely fusiform (Cohen’s *d* = −0.15, *p* = 0.0001), rostral middle frontal cortex (Cohen’s *d* = −0.13, *p* = 0.0001), superior frontal cortex (Cohen’s *d* = 0.13, *p* = 0.0007) and medial orbitofrontal cortex (Cohen’s *d* = −0.11, *p* = 0.001), were significantly associated with ASD after multiple testing correction (Fig. 2 and Supplementary Table 4). Overall, reduced leftward lateralization was observed in network measure HDs of the fusiform, rostral middle frontal and medial orbitofrontal cortex in ASD (Supplementary Tables 3 and 4). Superior frontal cortex showed reduced rightward asymmetry of both degree centrality and global efficiency HDs in ASD (Supplementary Tables 3 and 4). There were no significant associations between ASD and the HDs of the nodal clustering coefficient or nodal local efficiency after FDR correction (Fig. 2 and Supplementary Tables 5 and 6).

Further investigating the significant effects on asymmetry in unilateral analyses, the effects on degree centrality asymmetries of the fusiform and rostral middle frontal cortex, and nodal global efficiency asymmetries of the fusiform and medial orbitofrontal cortex, involved right-sided increases, thus resulting in reduced leftward topological asymmetries (Supplementary Table 7). For the effect on nodal global efficiency asymmetry of the rostral middle frontal cortex, bilateral increases were observed in ASD, but more so in the right than left hemisphere, consistent with reduced leftward lateralization in ASD individuals relative to controls. The effects on degree centrality and nodal global efficiency asymmetries of the superior frontal cortex involved bilateral decreases in ASD, but more so in the right hemisphere, consistent with reduced rightward asymmetry of these metrics in ASD (Supplementary Table 7).

**Fig. 3:**
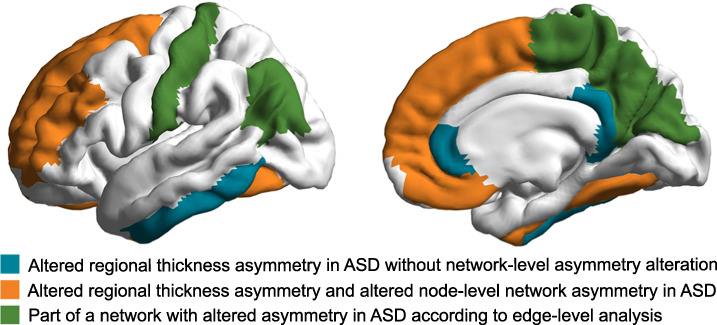
Regions with altered average network-level asymmetries in ASD compared to separate region-by-region testing. The color key is indicated in the figure. See the main text for the citation of the study that performed separate region-by-region testing.

All four regions that showed significant case-control differences in node-level asymmetry analysis were among seven that exhibited altered cortical thickness asymmetry in separate region-by-region testing in the previous ENIGMA-ASD study of asymmetry [17] the four regions were concentrated in the frontal lobe and fusiform cortex (Fig. 3).

### Clinical severity, medication, IQ, age, sex, and handedness

For the 7 network HDs that showed significant case-control differences in our main analysis, i.e. the degree centrality HDs of fusiform, rostral middle frontal and superior frontal cortex, and the nodal global efficiency HDs of fusiform, rostral middle frontal, superior frontal and medial orbitofrontal cortex, we found no significant associations with autism symptom severity (total ADOS [63] scores) (*p*s > 0.05; Supplementary Table 8). There also were no significant associations of current medication use with these metric HDs after FDR correction (Supplementary Table 9). Medication status showed a nominally significant association with the degree centrality HD of the fusiform (Cohen’s *d* = −0.22, unadjusted *p* = 0.04), and a marginal trend with fusiform nodal global efficiency HD (Cohen’s *d* = −0.19, unadjusted *p* = 0.06). There were no significant associations of IQ with the network HDs within ASD individuals (*p*s > 0.05; Supplementary Table 10). Age showed a significant positive association with the nodal global efficiency HD of the medial orbitofrontal cortex (*t* = 2.36, unadjusted *p* = 0.006; Supplementary Table 11). There were no significant associations between network HDs and sex (Supplementary Table 12) or handedness (Supplementary Table 13).

### Descriptive edge-level analysis

**Fig. 4:**
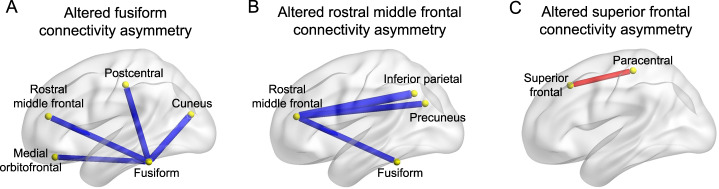
Altered asymmetry of connectivity linking to the nodes with significant alterations of degree centrality asymmetry in ASD. **a** Altered asymmetry of connectivity linked to the fusiform in ASD. **b** Altered asymmetry of connectivity linked to the rostral middle frontal cortex in ASD. **c** Altered asymmetry of connectivity linked to the superior frontal cortex in ASD. The yellow nodes indicate the brain regions. Red indicates a significant edge-level, reduced rightward asymmetry of connectivity in ASD compared to controls, and blue indicates an edge-level, reduced leftward asymmetry of connectivity in ASD compared to controls.

The degree centrality of each node provides a metric of its hemisphere-wide connectivity. For the three regions that showed significant associations between their degree centrality HDs and ASD in the main analysis, i.e., fusiform, rostral middle frontal and superior frontal cortex, we performed descriptive edge-level analysis of case-control associations. Four edges linked to the fusiform cortex showed significant associations with ASD after FDR correction, which linked to the rostral middle frontal (Cohen’s *d* = −0.12, *p* = 0.0004), cuneus (Cohen’s *d* = −0.14, *p* = 0.0005), medial orbitofrontal (Cohen’s *d* = −0.11, *p* = 0.002), and postcentral regions (Cohen’s *d* = −0.13, *p* = 0.0006; Fig. 4A and Supplementary Table 14). These edges all showed reduced leftward asymmetry in ASD relative to controls (Supplementary Table 14). A significant association was also observed between ASD and connectivity asymmetry between the rostral middle frontal and three other regions, which were the inferior parietal region (Cohen’s *d* = −0.13, *p* = 0.0004), fusiform (Cohen’s *d* = −0.12, *p* = 0.0004), and precuneus (Cohen’s *d* = −0.17, *p* < 0.0001) after FDR correction (Fig. 4B and Supplementary Table 15). All of these effects involved lower leftward asymmetry in ASD compared to controls. In addition, connectivity between the superior frontal and paracentral cortex showed a significant association with ASD (Cohen’s *d* = 0.12, *p* = 0.001; Fig. 4C and Supplementary Table 16). This connectivity showed lower rightward asymmetry in ASD compared to controls.

In total, among the nine regions with altered connectivity asymmetry in ASD according to edge-level analysis, four were among those associated with altered cortical thickness asymmetry as previously found in separate region-by-region testing in the ENIGMA ASD data [17] (Fig. 3). These four regions were the same as identified in node-level asymmetry analysis (above), i.e. concentrated in frontal and fusiform regions.

### Functional annotation of networks with altered lateralized connectivity in ASD

**Fig. 5:**
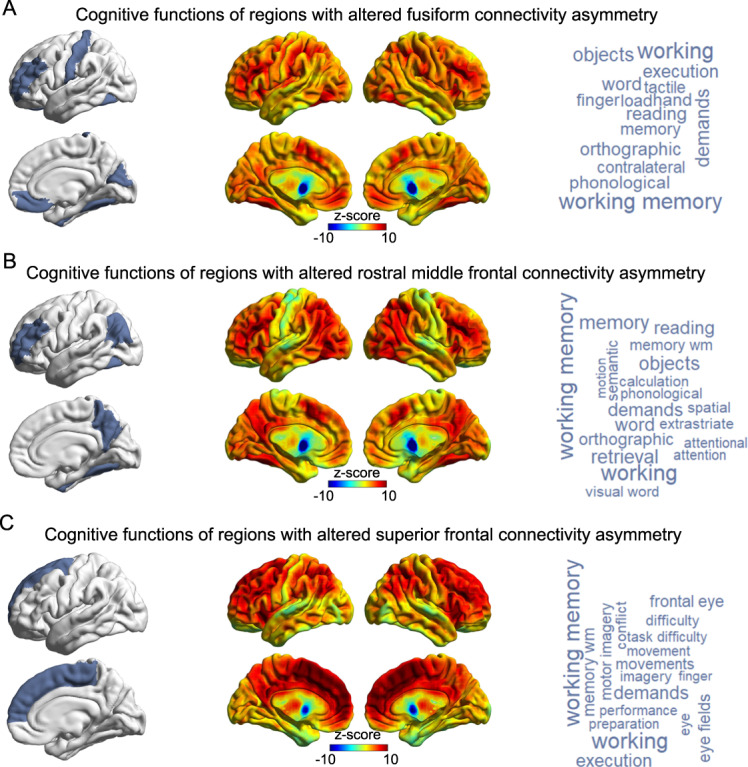
Cognitive functions associated with cortical regions showing altered connectivity asymmetry. Meta-analyzed fMRI data were used to functionally annotate cortical regions showing altered connectivity asymmetry with the fusiform (**a**), rostral middle frontal (**b**) or superior frontal (**c**) cortex. Left panels indicate the regions showing alterations of lateralized connectivity, which were used as input masks to the decoder function of Neurosynth (see Methods). Middle panels show the brain co-activation maps corresponding to the input masks. Right panels show the cognitive terms corresponding to the co-activation maps, in word-cloud plots. The font sizes of the cognitive terms indicate their map-wide correlations with the co-activation maps (correlation coefficients are in Supplementary Table 17).

The most prominently shared functional annotation for all three networks that showed associations of degree centrality asymmetry with ASD was “working memory” (Fig. 5 and Supplementary Table 17). However, each network also had additional cognitive annotations. Disrupted asymmetry of fusiform connectivity involved cortical regions that are especially active during executive control, reading and motor tasks (Fig. 5A and Supplementary Table 17). Regions with altered connectivity asymmetry linked to the rostral middle frontal cortex were associated with executive, reading and attention tasks (Fig. 5B and Supplementary Table 17). Finally, alteration of superior frontal connectivity asymmetry involved regions associated with executive and sensorimotor tasks (Fig. 5C and Supplementary Table 17).

### Sensitivity analyses

Across the defined range of sparsity thresholds (0.25–0.5), all of the associations remained significant between ASD and the asymmetries of degree centrality and nodal global efficiency, for the regions of fusiform, rostral middle frontal, superior frontal and medial orbitofrontal cortex (Supplementary Table 18); this indicated that the findings were robust to threshold selection.

After adding a non-linear age term to the linear mixed effects models, the effects of case-control status on the 7 affected network HDs remained significant and largely unaffected (Supplementary Table 19).

After controlling for global average cortical thickness, the same seven network HDs remained significantly associated with case-control status as in the main analysis (Supplementary Table 20).

## Discussion

### Altered average structural network asymmetry in ASD

We used a cortex-wide, graph-based approach to investigate differences of topological network asymmetries between individuals with ASD and unaffected controls across 43 datasets of the ENIGMA consortium’s ASD Working Group. We found significantly altered average asymmetries of topological network measures in individuals with ASD relative to controls, specifically involving nodes that comprised fusiform, rostral middle frontal, superior frontal and medial orbitofrontal cortex. The findings were largely, but not wholly, driven by a shift to greater randomization of right hemispheric network organization in ASD. Edge-level analysis from these nodes implicated structural covariance networks over prefrontal, parietal, posterior cingulate and paracentral cortical regions. Data-driven functional annotation, using meta-analyzed fMRI data, consistently identified working memory as a function that may be especially affected by network asymmetry alterations in ASD, consistent with executive function challenges characteristic of ASD [64–66].

The network-level findings provide a new understanding of the widespread, dispersed topography of altered average cortical thickness asymmetry in ASD, which was reported in a previous ENIGMA-ASD study based on separate region-by-region testing [17]. Specifically, out of seven regions that were previously shown to have altered average cortical thickness asymmetry in ASD [17], four were identified in the present study to be nodes of broader networks with altered average structural connectivity asymmetry. These four regions are notably concentrated in frontal and fusiform cortex (Fig. 3), which points to these regions in particular as important for ASD manifestation, while cingulate and inferior temporal regions with altered average thickness asymmetries were not identified as being involved in networks with altered topographic asymmetry. Rather, edge-level analysis from the frontal and fusiform nodes implicated mostly parietal regions (Fig. 3), which did not themselves show significant evidence for altered thickness asymmetry in separate region-by-region testing [17]. Thus, the altered average cortical thickness asymmetries in ASD can now be understood in terms of whether, and how, they are embedded within specific structural networks that show altered asymmetry in ASD, and with particular functional annotations of the affected networks (more on this below).

In general, many cognitive processes involve a degree of left-right hemispheric dominance in the healthy brain [7], so that the typical asymmetry pattern in the population is likely to be an optimal form of brain organization. It follows that alterations of network-level asymmetry may have functional consequences. As ASD is a childhood-onset disorder, and the majority of individuals in this study were children, the present findings provide further evidence that altered lateralized neurodevelopment is subtly disrupted in ASD. As noted in the Introduction, inter-regional co-variance at the macro-anatomical level may come about because synapses have mutually trophic and protective effects on pre- and post-synaptic neurons that connect spatially distant regions [26], or else synchronous neuronal firing may favor coordinated synaptogenesis and growth of highly connected regions [33, 34]. The network-level findings are also consistent with the hypothesis that inter-regional connections may propagate disrupted cortical thickness asymmetries, for example via aberrant neuronal signaling or disruption to neurotransmitter systems [26], among sets of spatially distant cortical regions–identified here specifically as frontal and fusiform regions. That is, intra-hemispheric topographic connectivity may contribute to shaping spatial patterns of cortical pathology in ASD. These findings therefore provide a possible explanation for some of the non-contiguous, average alterations of thickness asymmetry in ASD over the cortical surface.

The effect sizes in this study were small, with Cohen’s *d* ranging from −0.15 to 0.14. These findings indicate that case-control group average differences in structural network asymmetry are very subtle in ASD, and of similar magnitude to those reported in previous ENIGMA consortium studies of brain regional anatomy and asymmetry in ASD [17, 23]. Future studies may apply normative modeling [67] or clustering [68] approaches to identify subgroups of individuals with highly atypical structural network asymmetry, and these may constitute etiological subgroups of ASD. MRI-based regional cortical thickness measures are fairly crude biological readouts, affected by numerous possible underlying factors, including the degree of myelination [69], as well as the numbers and densities of different types of cells and dendritic processes [70–72]. Therefore it remains possible that subtly altered network asymmetry at the macro scale in ASD will be found to reflect more substantial alterations at finer levels of analysis. For example, neurite orientation dispersion and density imaging has been used to study grey matter microstructural asymmetries in vivo [73], or the ratio of T1w and T2w images in grey matter can be used to create an estimate of cortical myelin content [74]. Future *post mortem* studies of cortical histology and gene expression may also reveal microstructural and/or molecular alterations, but there is currently limited data available from homotopic regions of the two hemispheres, as many brain banks assign the left and right hemispheres into distinct storage and analysis protocols [75].

### Functional annotation of affected networks

Three specific cortical regions had node-level degree centrality asymmetries that were significantly altered in ASD: the fusiform, rostral middle frontal, and superior frontal cortices. Our meta-analyzed fMRI-based annotations implicated a range of functions mapping to each of the affected networks involving these regions (Fig. 5), which included working memory and other executive function-related annotations in common across the networks, but also language-related, reading-related, and sensorimotor annotations. Language delay is a common feature of ASD [5, 6], and the disorder is also associated with reduced left-hemisphere language dominance [12]. Numerous reports based on behavioral, neurophysiological, neuroimaging or histopathological data have also reported atypical motor system development in ASD [76]. Our findings may therefore indicate that alterations of specific right-hemisphere structural networks underlie some of the language- and motor-related deficits in ASD. These functional annotations, that were based on meta-analyzed fMRI data from other cohorts, motivate future studies of brain-behavior correlations using neuroimaging and behavior data from the same affected individuals.

The fusiform gyrus is especially known to show right-lateralized activation in response to face-related perception [77–80], which is important in social interactions. Reduced rightward functional asymmetry for face processing has been associated with ASD [81], so face processing may be one aspect of cognition that is disrupted by increased randomization of a right-hemispheric structural network that includes the fusiform gyrus. The rostral middle frontal cortex (dorsolateral prefrontal cortex), has been proposed to act as a coordinating hub in cognitive control tasks, as part of a frontal-parietal network [82]. This region has been shown to be abnormally active in the left hemisphere in ASD relative to typically developing controls in a recent meta-analysis of cognitive control tasks [83]. Resting-state fMRI data have also suggested a rightward shift in asymmetry of executive control networks in ASD [84]. Moreover, white matter network analysis has suggested that individuals with ASD exhibit a greater age-related increase in global efficiency involving the right dorsolateral prefrontal cortex than typically developing controls [85]. The superior frontal cortex is known as a core region of the default mode network, which can show altered functional asymmetry in ASD [84]. Abnormal lateralization of functional connectivity between the superior frontal gyrus and temporal cortex has also been reported in ASD, and associated with language and social deficits [13]. Our findings further support altered lateralization of superior frontal cortex connectivity in ASD, demonstrated here on a structural level.

For the medial orbitofrontal cortex, there was a significant association of its nodal global efficiency asymmetry with ASD, but no significant association with its degree centrality asymmetry, and we therefore did not include it in fMRI-based annotation and edge-level analysis. The medial orbitofrontal cortex was the only cortical region to show altered asymmetry of both cortical thickness and surface area in individuals with ASD, in a previous ENIGMA consortium study that tested it separately region-by-region (not in a network context) [17]. Another study found that alterations in structural covariance between inferior frontal cortex and the left orbitofrontal cortex was modulated by language ability within ASD individuals [40], suggesting a possible contribution of the orbitofrontal cortex to communication deficits in ASD.

### Heterogeneity and clinical features

Within ASD individuals, we found no associations of the affected network asymmetry metrics with autism symptom severity, psychiatric medication usage, IQ, sex or handedness. Age showed an association with one network metric HD, i.e., the nodal global efficiency HD of the medial orbitofrontal cortex, but apart from this single effect, we were unable to link structural network asymmetries to the within-case phenotypic variables available in the current study. Age and sex were also generally of little significance as covariate effects in the main case-control analyses (Supplementary Tables 1–6), likely because these effects are mostly bilateral in nature, with limited impact on hemispheric differences. Deeper phenotyping may be needed to understand the relevance of structural connectivity asymmetry alterations in terms of clinical heterogeneity [86, 87]. For example, only total ADOS scores were available through the consortium (rather than subscores that reflect different behavioral dimensions) [63], and data on medication usage and comorbidities were limited to relatively small subsets of the overall data (see Methods). Future longitudinal studies may help to characterize atypical developmental trajectories of asymmetry patterns in ASD, and capture causal and dynamic processes of structural asymmetry alterations over the course of the disorder. It is also possible that altered structural connectivity will not map onto any identifiable symptom domains of ASD, but rather reflects a shared susceptibility mechanism across various individuals with heterogeneous presentations of ASD, and potentially other diagnoses too.

## Limitations

As mentioned above, the ENIGMA-ASD data generally lack consistent, deep phenotyping to better understand the impacts of clinical heterogeneity. This is an inevitable consequence of making use of legacy datasets which were initially collected and conceived as separate studies. In addition, there is little consistent multi-modal MRI or longitudinal data available across the datasets. This means that the structural network alterations identified here, on the basis of regional cortical thickness asymmetries, cannot be further supported in these individuals by data on e.g. white matter tracts, functional connectivity or intra-individual maturational changes.

The present study was framed around asymmetries of intra-hemispheric networks, and did not consider inter-hemispheric connectivity. This permitted consistent use of the same network definitions through all stages of analysis, from the whole-hemisphere level through to node-level and edge-level analyses. Including inter-hemispheric connectivity in network construction would result in single network-level parameters (gamma, lambda, sigma) being measured for the whole brain, rather than separately by hemisphere–the latter is a prerequisite for asymmetry analysis at the whole-hemisphere level. At the nodal level, including a mixture of intra- and inter-hemispheric connectivity would mean including edges between homotopic regions in the two hemispheres, which cannot contribute to asymmetry. This would reduce hemispheric asymmetries in the measured node-level connectivity, and complicate subsequent edge-level analysis and interpretation for the nodes that showed altered asymmetry of degree centrality in individuals with ASD. Investigating networks with only intra-hemispheric connectivity therefore supported coherence and comparability across all analyses in the present study. Future studies of asymmetry may consider inter-hemispheric edges specifically for node level analysis.

Here we focused on four graph-level metrics that have been applied previously in studies of cortical thickness-based structural networks in brain health and disease (see Introduction and Methods). Between them, these metrics directly describe global and local network connectivity. More broadly, graph theory offers a large range of additional metrics to explore in future studies, especially metrics which first require defining network modules [88]. However, this can involve arbitrary thresholds, while modules may not be consistent across datasets, and increased multiple testing can become an issue.

In this study we used the HD (Left-Right) to quantify asymmetries of topological properties. The HD does not adjust for the bilateral sum or average in the same way as the more usual asymmetry index (AI), e.g. calculated as (Left-Right)/(Left+Right). This means that the magnitude of the HD partly relates to the magnitude of the bilateral measures used in its calculation. However, the HD is more robust than the AI for the type of Left and Right variables used in the present study, as low values of Left+Right can result in extreme values of a classic AI–in principle such extreme values could both mask or inflate case-control differences. Reassuringly, controlling for the global average cortical thickness of each individual produced the same set of significant findings as the main analysis. Therefore the results can be understood in terms of hemispheric asymmetry rather than cortex-wide effects, to which bilateral regional effects are related.

## Summary conclusion

In conclusion, this consortium study identified small group-average differences between ASD individuals and unaffected controls in specific aspects of the asymmetry of hemispheric structural connectivity networks. The affected nodes were specifically in frontal and fusiform regions, and the implicated networks mapped most consistently to working memory as a function that depends on the implicated regions. These findings help to elucidate altered cortical thickness asymmetry in ASD in terms of hemispheric network architecture, and suggest that some specific neurodevelopmental alterations of brain asymmetry in ASD may propagate via structural connectivity.

## Supplementary information

Supplementary Methods.

Supplementary Tables.

## Data Availability

This study made use of 43 separate data sets collected around the world, under a variety of different consent procedures and regulatory bodies, during the past 25 years. Requests to access the data sets will be considered in relation to the relevant consents, rules and regulations, and can be made via the ENIGMA consortium’s ASD working group http://enigma.ini.usc.edu/ongoing/enigma-asd-working-group/

## Code availability

This study used openly available software and codes, specifically Brain Connectivity Toolbox (https://www.nitrc.org/projects/bct) and GRETNA (https://www.nitrc.org/projects/gretna/).
