# Supplementary material for: Subtly altered topological asymmetry of brain structural covariance networks in autism spectrum disorder across 43 datasets from the ENIGMA consortium
Source: Mol Psychiatry. 2022 Feb 8;27(4):2114–25. doi: 10.1038/s41380-022-01452-7 (PMC9126820; doi:10.1038/s41380-022-01452-7)
Supplement: Supplementary file 1 — Supplementary Methods. [file 41380_2022_1452_MOESM1_ESM.docx]

**Supplementary Information**

**Sha *et al*., Subtly altered topological asymmetry of brain structural covariance networks in autism spectrum disorder across 43 datasets from the ENIGMA consortium**

**Contents:**

**Supplementary Methods -** Pages 2-6

**Supplementary Tables -** See separate spreadsheet file containing 20 supplementary tables, one table per worksheet.

**Supplementary Methods**

**Assessment of participants**

Datasets were collected as separate studies between 1994 and 2013, when DSM-IV and DSM-IV-TR were the common classiﬁcation systems. For each dataset, all subjects were diagnosed by a clinically experienced and board certiﬁed physician/psychiatrist/psychologist. Data on DSM-IV subtypes of ASD were not collated by the ENIGMA ASD working group.

Total scores from the Autism Diagnostic Observation Schedule-Generic (ADOS) were available for 704 individuals with ASD. The presence or absence of comorbid conditions had been recorded for 200 individuals with ASD, and there were 43 affected individuals diagnosed with at least one other co-occurring condition (e.g., attention deficit hyperactivity disorder, obsessive-compulsive disorder, depression, anxiety and/or Tourette's syndrome). Data on the presence or absence of medication use at the time of scanning (i.e., current use of psychiatric treatment drugs prescribed for ASD or comorbid psychiatric conditions) were available for 612 individuals with ASD, of whom 173 were current users. Data on IQ were available for 1,210 of the ASD individuals. Cases from the entire ASD spectrum were included, but only 61 cases had IQ<70 (cases: mean IQ=103.49, SD=19.74, min=34, max=149). Binary categorical data on handedness were available for 599 ASD individuals (551 right-handed, 48 left-handed).

There were different assessment and recruitment processes for controls across the datasets, but the overwhelming majority were typically developing at the time of scanning, and no controls met criteria for a diagnosis of ASD. Only 18 controls had IQ<70. In these individuals the exclusion of an ASD diagnosis was performed by a senior child psychiatrist/physician. All eighteen of these were from the FSM data set and were clinically diagnosed with idiopathic intellectual disability. Amongst all 1,303 controls with IQ data, the mean IQ was 111.75, SD=14.73, min=31, max=149.

**MRI processing and quality control**

Processing and quality control followed the ENIGMA consortium protocol (<http://enigma.ini.usc.edu/protocols/imaging-protocols>). The default ‘recon-all’ pipeline of FreeSurfer was used, which incorporates renormalization. Parcellations of cortical regions were visually inspected following the standardized ENIGMA quality control protocol (<http://enigma.ini.usc.edu/protocols/imaging-protocols>). Briefly, web pages were generated with snapshots from internal slices, as well as external views of the segmentations from different angles. For subcortical structures, the protocol also consisted of visually checking individual images, plotted from a set of internal slices. Values derived from incorrectly labelled structures were excluded. Furthermore, any data points exceeding 1.5 times the interquartile range, as defined per site and diagnostic group, were visually inspected, and any errors resulted in excluded values.

Specifically for the present study, we also excluded any individuals with missing thickness data for at least one cortical region, as the analyses required all regions for comparability of networks across individuals. We also excluded datasets with fewer than 15 controls, as variation within the control group of each dataset is important for calculating intra-individual covariance (see main text). These steps resulted in 43 datasets being included in the present study, with the sample numbers given in the main text.

**Intra-individual structural covariance and network properties**

See the main Methods text for references that introduced and/or previously applied the following formulae in cortical thickness-based analysis of structural network connectivity.

The formula for calculating structural covariance was as follows:

$${ISC}_{k}\left( i,j \right)=\frac{1}{exp(\frac{{({CT}_{k}\left( i \right)-{CT}_{k}(j))}^{2}}{2\times({CT}_{SD}\left( i \right)+{CT}_{SD}(j))})}$$

where ${ISC}_{k}(i,j)$ represents the intra-individual structural covariance between region *i* and region *j* in individual *k.* ${CT}_{k}(i)$ and ${CT}_{k}(j)$ represent the cortical thicknesses of regions *i* and *j* in individual *k*. ${CT}_{SD}(i)$ represents the standard deviation of cortical thickness in region *i* across all control individuals in a given dataset.

Applying a sparsity threshold S=0.4 retained network connectedness, such that at least 88% of nodes (30 nodes) remained connected with at least one other node in all left- and right-hemisphere networks in all individuals (i.e., a maximum number of four isolated nodes in any network). Small-world organization was also retained (minimum small-worldness scalar σ=1.0001 in any network, see below). This approach ensured that all left- and right-hemisphere networks, in all individuals, had the same number of nodes (34) and edges (224), and enabled us to perform subsequent analyses with reference only to relatively high-level, reliable connectivity.

Small-worldness can be quantified by the clustering coefficient and shortest path length. Based on these measures, a network can be described as regular, random or small-world. A regular network is characterized by a high clustering coefficient and high shortest path length, indicating high local specialization (high local efficiency) and low global integration (low global efficiency). In contrast, a random network has a low clustering coefficient and low shortest path length, corresponding to low local specialization (low local efficiency) and high global integration (high global efficiency). In general, human brain networks are organized in an optimized, small-world fashion, with an intermediate balance between regular and random properties, i.e., a large number of short-range connections coexist with a smaller number of long-range connections.

The clustering coefficient *c_i_* of the node *i* was defined as

$$c_{i}=\frac{E_{i}}{k_{i}(k_{i}-1)/2}$$

where $E_{i}$ represents the number of existing edges among the neighbors of node *i*. $k_{i}$ denotes the actual number of neighbors of node *i*, thus the denominator quantiﬁes the number of all possible edges among the neighboring nodes. The clustering coefﬁcient *C* of a whole hemispheric network was then deﬁned as the mean clustering coefﬁcient across all nodes in that network, separately per individual and hemisphere.

The shortest path length $l_{i}$ of a node *i* was deﬁned as

$$l_{i}=\frac{1}{N-1}\sum_{i\neq j} min\left\{ l_{ij} \right\}$$

where $\min\left\{ l_{ij} \right\}$ represents the shortest path length between node *i* and *j*. *N* represents the number of nodes in the network. The shortest path length *L* of a network was then deﬁned as the mean shortest path length between any pair of nodes in the network, calculated separately per individual and hemisphere.

Next, we generated 100 random networks with the same number of nodes (34), edges (224), and degree distribution as the real networks to calculate a normalized clustering coefﬁcient γ=*C*/*C*_rand_ and normalized shortest path length λ=*L*/*L*_rand_, in which *C*_rand_ and *L*_rand_ were deﬁned as the mean clustering coefﬁcient and mean shortest path length across randomly generated networks. The small-world index σ was calculated as σ=γ/λ, which should be greater than 1 in small-world networks, and whose minimum value was 1.0001 across the networks of all individuals and hemispheres (see above).

The degree centrality of node *i* was defined as the sum of all existing edges between that node and all other nodes in the network, reflecting the importance of that node in network information communication.

The global efficiency of node *i* ($E_{glob}$) indexes information transfer from itself to all other nodes in the entire network, computed as the reciprocal of the shortest path length $l_{i}$ :

$$E_{glob}=\frac{1}{N-1}\sum_{i\neq j\in G} \frac{1}{L_{ij}}$$

where $L_{ij}$ is the shortest path length between node *i* and node *j* in network *G*. *N* is the number of nodes in the network.

The clustering coefficient measures the extent of local density of connections for a given node (see the formula further above, where node-level clustering coefficients were calculated as a step towards the hemisphere-level clustering coefficient).

The local efficiency of node *i* ($E_{loc}$) corresponds to the efficiency of information flow within the local environment, which is defined as

$$E_{loc}=\frac{1}{N}\sum_{i\in G} E_{glob}(G_{i})$$

where $G_{i}$ represents the subgraph composed of the nearest neighbors of node *i*.

**Cohen’s d calculation**

We calculated Cohen’s d from t values using the following formula

$$d=\frac{t\times(n_{1}+n_{2})}{\sqrt{n_{1}\times n_{2}}\times\sqrt{df}}$$

where $n_{1}$ and $n_{2}$are the numbers of cases and controls, and *df* represents the degrees of freedom. $df=obs-(x_{1}+x_{2})$, where *obs* equals the number of observations, $x_{1}$ the number of groups and $x_{2}$ the number of factors in the model.

**Permutation procedure for case-control analysis**

Permutations (N=10,000) were used to test the significance of each case-control diagnosis effect on each HD metric, by randomly assigning the diagnosis labels across individuals, separately within each dataset while maintaining the same numbers of cases and controls within each dataset, prior to mega-analysis across datasets. Shuffling was carried out within datasets separately because intra-individual covariance was calculated with reference to the variance in matched controls (see above). The empirical p value was obtained for each diagnosis effect on each HD metric by counting the number of unsigned *t* values in the permutation analysis that were greater than the unsigned *t* value for the data with the real case-control labels, and dividing that number by the total number of permutations (N=10,000).

**Associations with ASD severity, medication, IQ, age, sex or handedness**

Autism severity was based on the total ADOS scores of ASD individuals (N=704):

$$HD=ADOS+age+sex+random (dataset)$$

The presence/absence of current psychiatric medication use was coded as a binary predictor variable (0=no medication, 2=medication) (N=612)

$$HD=medication+age+sex+random (dataset)$$

IQ was tested for association with HDs within the 1,210 ASD individuals with IQ data. IQ was coded as a continuous predictor variable:

$$HD=IQ+age+sex+random (dataset)$$

Age and sex were tested for associations with HDs within 1,455 ASD individuals (age as a continuous variable, sex as a binary variable:

$$HD=age+sex+random (dataset)$$

Handedness was tested for association with HDs within 599 ASD individuals that had data on this trait. Handedness was coded as a binary predictor variable (1=right handedness, 2=left handedness).

$$HD=handedness+age+sex+random (dataset)$$

For each of these analyses, permutations (N=10,000) were used to test the significance of each effect of interest, by shuffling the relevant variable (either ADOS scores, medication use, IQ, age, sex or handedness) across case individuals, separately within each dataset, prior to mega-analysis across datasets. *P* values were obtained by counting the number of unsigned *t* values in the permutation tests that were greater than the actual unsigned *t* values for the real data, and dividing by the total number of permutations (N=10,000) for each separate analysis. *P* values were FDR-corrected at 0.05 for multiple testing over the number of network HDs that showed significant effects in the main analysis (i.e., seven network HDs, see Results). We did not additionally correct for multiple testing over the six variables of interest (ADOS scores, medication use, IQ, age, sex or handedness) as this was an exploratory analysis to describe how each case heterogeneity variable might impact the network HDs.

**Sensitivity analyses**

To assess robustness with respect to the sparsity threshold 0.4 that was used in the main analysis, we repeated the analyses under varying sparsity thresholds ranging from 0.25 to 0.5 (with an interval of 0.01). At the lowest threshold, a minimum 79% of nodes (27 nodes) were connected to at least one other node in all hemispheric networks in all individuals (maximum seven unconnected nodes out of 34). We then computed the area under the curve for each network metric HD over the range of sparsity thresholds. The area under the curve for a given network HD *Y*, calculated over the sparsity threshold range of S_1_ (0.25) to S_n_ (0.5) with interval of ΔS (0.01), was computed as

$$Y^{area under the curve}=\sum_{k=1}^{n-1} [Y\left( S_{k} \right)+Y(S_{k+1})]\times\Delta S/2$$

Case-control differences in the area under the curve for each network metric HD were then tested separately using the same mixed effects random-intercept model as the main analysis:

$$HD=diagnosis+age+sex+random (dataset)$$

To assess whether non-linear age could have an impact on case-control differences of network HDs, we added a non-linear age term ‘zage^2^’, i.e., (age-mean_age)^2^ as a fixed effect in the linear mixed effects model to test each association:

$$HD=diagnosis+age+{zage}^{2}+sex+random (dataset)$$

Permutations (N=10,000) were used as in the main analysis, to determine the empirical significance of case-control differences for each network HD metric.
